# Case Report: Metronidazole-induced encephalopathy in a patient with acute myeloid leukemia type 2 and a literature review of the pediatric case reports

**DOI:** 10.3389/fped.2025.1656227

**Published:** 2025-09-29

**Authors:** Chunyan Zhao, Qingping Zhang, Meijiao Zhang, Jie Zhang, Xinhua Bao

**Affiliations:** Department of Pediatrics, Peking University First Hospital, Beijing, China

**Keywords:** metronidazole-induced encephalopathy, metronidazole, encephalopathy, MRI, pediatric

## Abstract

**Background:**

Metronidazole exerts neurotoxic effects on the central nervous system, leading to rare pediatric cases of metronidazole-induced encephalopathy (MIE).

**Methods:**

This study reports a pediatric patient with MIE. The patient's clinical and neuroimaging data was collected and analyzed. A literature review of pediatric MIE cases reported up to June 2025 was conducted using PubMed, Embase, Web of Science, Cochrane Library, WanFang, and CNKI databases.

**Results:**

A 13-year-old female developed MIE after two courses of metronidazole with cumulative doses of 31.2 g and 37.6 g. Clinical manifestations included altered mental status, ophthalmoplegia, dysarthria, dysphagia, involuntary movements, dystonia, urinary retention, muscle weakness, and absent tendon reflexes. Brain MRI revealed T2W/FLAIR and DWI hyperintensities symmetrically involving the dentate nuclei, splenium of corpus callosum, basal ganglia, thalamus, midbrain, and pons. After discontinuing metronidazole and vitamin B1 therapy, the patient's symptoms gradually improved, though extrapyramidal symptoms persisted. Repeated brain MRI at 10 days after cessation showed lesions partially resolved. Literature review identified 18 pediatric MIE cases, totaling 19 cases including our case. Except for one infant case presenting with intermittent hypothermia, bradycardia, growth retardation, reduced activity, muscle weakness and hypotonia, the predominant neurological symptoms in the other 18 cases were ataxia (11/18), altered mental status (8/18), dysarthria (7/18), and epileptic seizures (5/18). Symmetric brain MRI lesions involving dentate nuclei (13/17), splenium of corpus callosum (9/17), midbrain (6/17), pons (5/17). Most patients (17/19) achieved complete clinical resolution and brain MRI improvement after metronidazole withdrawal.

**Conclusion:**

Pediatric MIE is rare and manifests with widespread neurological involvement, characterized by cerebellar dysfunction, altered mental status, and seizures. Brain MRI typically reveals symmetrical lesions in the dentate nuclei, splenium of corpus callosum, and midbrain. Timely drug discontinuation is critical for treatment, and most cases are reversible, though a few may leave neurological sequelae.

## 1 Introduction

Metronidazole-induced encephalopathy (MIE) is a condition secondary to metronidazole's neurotoxic effects on the central nervous system. It typically manifests as cerebellar dysfunction, altered mental status, seizures, and other neurological symptoms. Radiographically, MIE is frequently characterized by symmetrical involvement of the cerebellar dentate nuclei, splenium of the corpus callosum, and the dorsal brainstem (1, 2). The incidence of MIE remains unknown. Literature reports indicate that MIE is predominantly observed in adults, with pediatric cases being exceedingly rare, partially due to the relatively infrequent clinical use of metronidazole in children. This article reports a case of MIE in a 13-year-old female with acute myeloid leukemia subtype 2 (AML-M2) who underwent two allogeneic hematopoietic stem cell transplantations (Allo-HSCTs). MIE occurred after prolonged metronidazole administration for Clostridioides difficile associated diarrhea (CDAD). Additionally, we provide a review of published pediatric MIE cases in the literature. This report aims to enhance awareness and vigilance regarding MIE among pediatric clinicians.

## 2 Methods

### 2.1 Patient and clinical study

A 13-year-old female developed MIE following two prolonged courses of metronidazole therapy. We retrospectively collected and analyzed her clinical data, including the duration and dosage of metronidazole treatment, clinical manifestations, ancillary examinations, and outcome. This study was approved by the Medical Ethics Committee of Peking University First Hospital (2025-1149).

### 2.2 Literature review

A literature search was performed in PubMed, Embase, Web of Science, Cochrane Library, WanFang, and CNKI databases using the keywords: “Metronidazole-induced encephalopathy,” “Metronidazole,” “encephalopathy,” and “children.” The date of the last search was June 20, 2025. Case data were extracted using a standardized collection form with predefined fields capturing: indication for metronidazole therapy, administration route, treatment duration and cumulative dosage, clinical manifestations, brain MRI lesions, therapeutic interventions, and outcomes.

## 3 Results

### 3.1 Clinical description and investigation

**Figure 1:**
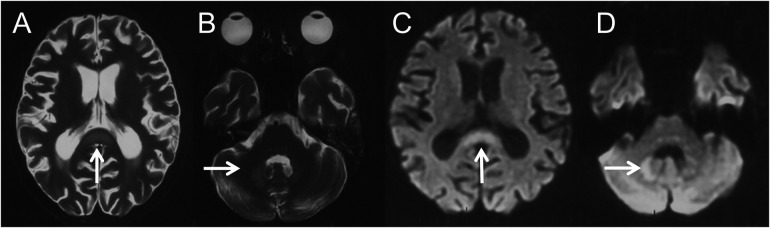
Four days after the onset of MIE, brain MRI revealed symmetric lesions with hyperintensities on axial T2W **(A,B)** and DWI **(C,D)** images over the splenium of corpus callosum **(A,C**, thin arrows**)** and dentate nucleus **(B,D**, thin arrows**)**.

A 13-year-old female was diagnosed with AML-M2 at the age of 10. She underwent two Allo-HSCTs, 2 years and 5 months before the current presentation, respectively. After the second transplant, she developed intermittent diarrhea with occasional pseudomembranous stools, prompting clinical suspicion of CDAD. Intravenous metronidazole therapy was initiated at 7.5 mg/kg every 6 hours and continued for 39 days (cumulative dose: 31.2 g). Forty-four days after discontinuation, recurrent diarrhea prompted re-initiation of metronidazole at the same daily dose. After 47 days of continuous metronidazole therapy (cumulative dosage 37.6 g), she developed irritability, dysarthria, and involuntary movements including tongue protrusion, blinking, and grimacing. Brain magnetic resonance imaging (MRI) performed 4 days after symptom onset demonstrated cerebral atrophy and symmetrical lesions in the splenium of the corpus callosum and dentate nuclei with T1-weighted (T1W) hypointensity and T2-weighted (T2W)/fluid-attenuated inversion recovery (FLAIR)/diffusion-weighted imaging (DWI) hyperintensity (Figure 1). Brain MRI prior to the first metronidazole course was normal. Electroencephalography revealed a fast background rhythm with scattered multifocal sharp waves. Cerebrospinal fluid (CSF) analysis was normal. Laboratory tests indicated alanine aminotransferase 45 IU/L (normal range 9–50 IU/L), aspartate aminotransferase 64 IU/L (15–40 IU/L), total bilirubin 57.4 μmol/L (1.7–20 μmol/L), and direct bilirubin 29.4 μmol/L (0–6 μmol/L). The symptoms deteriorated gradually with lethargy, aphasia, dysphagia, dystonia, urinary retention, external ophthalmoplegia and chemosis.

**Figure 2:**
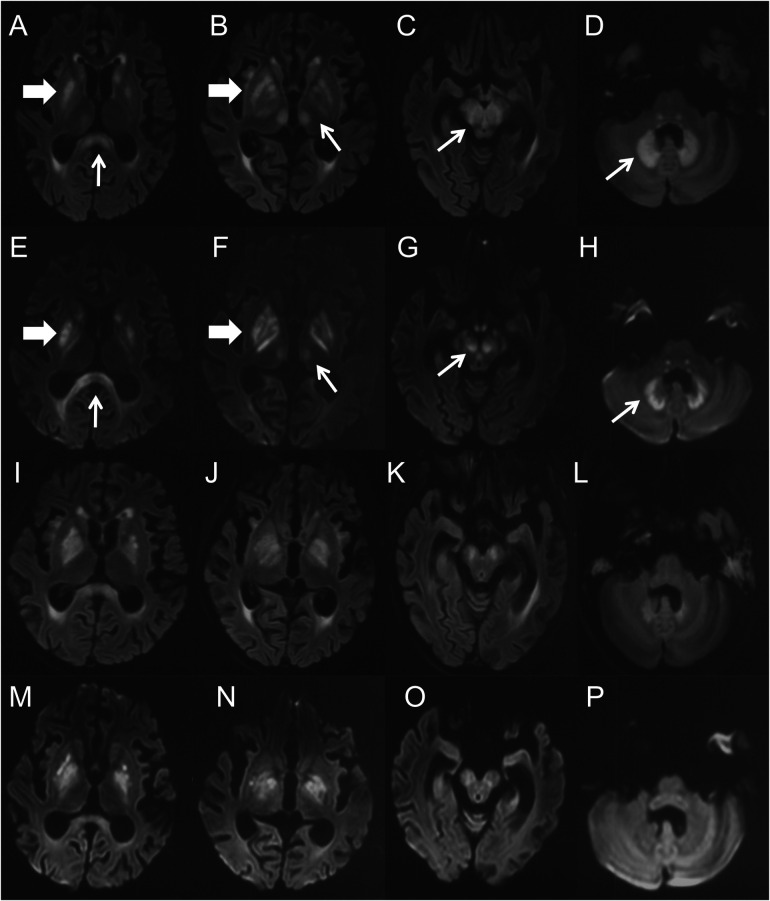
**(A–H)** were the brain MRI performed 14 days after the onset of MIE. **(A–D)** were the axial FLAIR images, **(E–H)** were the DWI images. Symmetric hyperintensity lesions presented over the splenium of corpus callosum **(A,E**, thin arrows**)**, basal ganglia **(A,B,E,F**, thick arrows**)**, thalamus **(B,F**, thin arrows**)**, midbrain **(C,G**, thin arrows**)** and dentate nucleus **(D,H**, thin arrows**)**. **(I–P)** were the brain MRI performed 22 days (10 days after drug withdrawal) after the onset of MIE. **(I–L)** were the axial FLAIR images, and **(M–P)** were the DWI images. They demonstrated complete (thalamus) or partial resolution (others) of the lesions.

**Figure 3:**
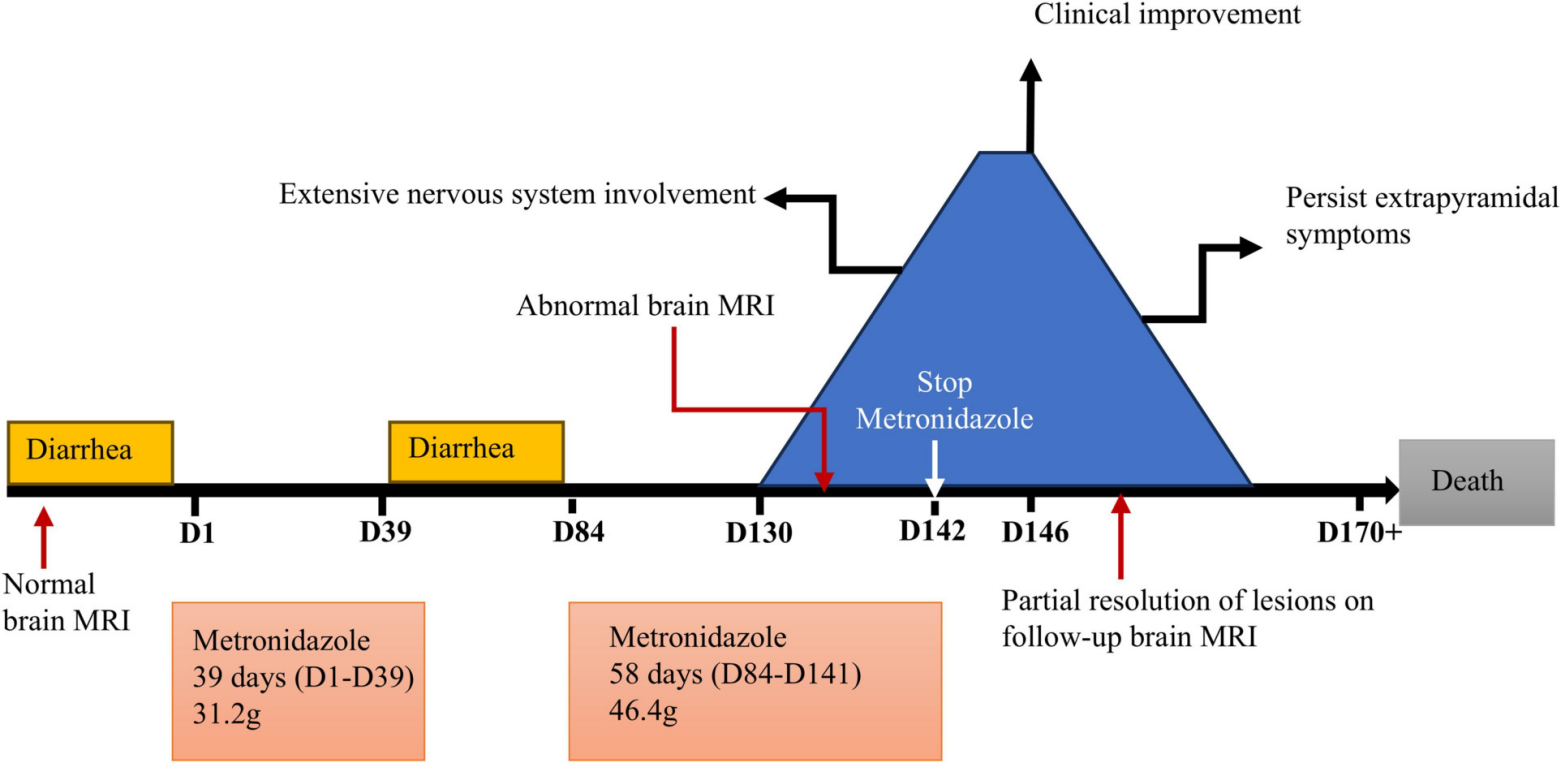
Patient's clinical course.

Ten days after the neurological symptom onset, she was transferred to our hospital. Based on typical clinical manifestations and characteristic brain MRI lesions, MIE was diagnosed. Metronidazole was immediately discontinued, and vitamin B1 supplementation was initiated. At this point, metronidazole had been administered for 58 days, with a cumulative dose of 46.4 g. Two days after discontinuation, a repeat brain MRI showed the lesions became more diffuse in the regions of basal ganglia, inferior colliculus of quadrigeminal bodies, midbrain, pons, thalamus and paraventricular white matter, in addition to splenium of corpus callosum and dentate nucleus (Figures 2A–H). Clinical improvement emerged four days after discontinuation with heightened alertness and responsiveness to simple verbal commands. A follow-up brain MRI 10 days later showed partial resolution of lesions (Figures 2I–P). Eleven days after discontinuation, the patient's ocular movements returned to normal, but extrapyramidal symptoms persisted. Regrettably, approximately one month after metronidazole withdrawal, the patient succumbed to acute gastrointestinal hemorrhage secondary to graft-vs.-host disease (GVHD) (Figure 3).

### 3.2 Literature review

A total of 18 articles were retrieved from PubMed, Embase, Web of Science, Cochrane Library, WanFang, and CNKI databases, reporting 18 pediatric cases of MIE (3–21) (Table 1). Combined with the case reported in this study, a total of 19 pediatric MIE cases have been documented to date. Ten cases originated from the United States, with the remainder reported from China, India, Qatar, Poland, Iran, Italy, and Turkey. Among the 19 patients, 15 were males (79%) and 4 were females (21%), with the age of onset ranging from 36 days to 18 years (median: 14 years). Indications for metronidazole therapy included gastrointestinal infection treatment or prophylaxis (17/19), central nervous system infection (1/19), and intrauterine fetal exposure (1/19). Administration routes encompassed intravenous, oral, and rectal. Duration of metronidazole use preceding MIE onset was reported in 14 patients, ranging from 22 hours to 3 years (median 28 days), while cumulative dosage data were available for 8 patients, ranging from 3 to 1,378.8 g (median 38.6 g). With the exception of a 36-day-old infant presenting with intermittent hypothermia, bradycardia, growth retardation, reduced activity, and decreased muscle strength and tone, the clinical manifestations in the remaining 18 patients included ataxia (11/18), altered mental status (8/18), dysarthria (7/18), seizures (5/18), dizziness/vertigo (4/18), visual impairment (3/18), dysphagia (3/18), extrapyramidal symptoms (2/18), hearing loss (1/18), urinary retention (1/18), and external ophthalmoplegia (1/18). Peripheral neuropathy, manifested as muscle weakness and/or paresthesia, was observed in 5 patients. Brain MRI results of 18 patients were reported, with 1 patient showing normal findings. The remaining 17 patients had symmetric hyperintensities on T2-weighted and FLAIR sequences, primarily involving the dentate nucleus (13/17), splenium of the corpus callosum (9/17), midbrain (6/17), pons (5/17), cerebral white matter (5/17), medulla oblongata (4/17), basal ganglia (4/17), thalamus (2/17), cerebellar white matter (2/17), cerebellar peduncle (1/17), cervical spinal cord (1/17), and optic tract (1/17). DWI/ADC performed in 11 patients revealed restricted diffusion in 8 patients, predominantly in the splenium of the corpus callosum, with variable high or low signal intensities on the ADC sequences. Twelve patients underwent follow-up brain MRI after metronidazole discontinuation. Nine patients were scanned 4 days to 7 months (median 40 days) after metronidazole discontinuation, while 3 patients did not report the follow-up time. Complete resolution of abnormal signals was observed in 6 patients, near complete resolution in 2 patients, improvement in 3 patients, and residual abnormal signals with cystic changes in the basal ganglia in one patient. All patients discontinued metronidazole upon MIE suspicion. Adjunctive therapies included plasma exchange in one patient, coenzyme Q10 administration in one patient, and thiamine supplementation in one patient. Seventeen (17/19) patients achieved complete resolution of clinical symptoms and recovered rapidly to baseline function. Two patients exhibited residual extrapyramidal symptoms, one of whom succumbed to complication of the underlying disease.

**Table 1:** Clinical characteristics of children with metronidazole-induced encephalopathy.

Year, country	Age (years, sex)	Indications for treatment	Routes of metronidazole	Cumulative dose to first CNS symptom (g)	Days to first CNS symptom	Symptoms and clinical findings	Lesion sites on MRI	Days from cessation of metronidazole to follow-up MRI	Follow-up MRI	Outcome
Our study, China	13/F	Clostridioides difficile-associated diarrhea	IV	68.8	86 days	Altered mental status, dysarthria, dysphagia, external ophthalmoplegia, extrapyramidal symptoms, urinary retention, peripheral neuropathy	Dentate nucleus, splenium of the corpus callosum, midbrain, pons, basal ganglia, thalamus, cerebral white matter	10 days	Improvement	Residual extrapyramidal symptoms, and succumbed to complication of the underlying disease
2025, Qatar (4)	18/M	Recurrent C. difficile colitis	PO	>400	3 years	Ataxia, dizziness, dysarthria	Dentate nucleus, splenium of the corpus callosum, midbrain, pons	1 months	Resolution	Resolution
2024, India (3)	14/F	Amebic liver abscess	IV	25.2	4 weeks	Ataxia, altered mental status	Dentate nucleus, midbrain, pons	6 weeks	Resolution	Resolution
2022, India (5)	11/M	Acute gastroenteritis	IV	3	2 days	Altered mental status, dysarthria	Splenium of the corpus callosum	4 weeks	Resolution	Resolution
2022, Poland (6)	16/F	Crohn's disease with perianal fistulas and abscesses	PO, IV, PR	52	28 days	Ataxia, dysarthria, dysphagia, peripheral neuropathy	Dentate nucleus, splenium of the corpus callosum, cerebellar white matte	NA	NA	Resolution
2021, USA (8)	12/M	Crohn's disease with chronic Clostridium difficile infection	NA	NA	75 days	Vertigo, ataxia	Dentate nucleus	NA	NA	Resolution
2021, USA (9)	14/M	Clostridium difficile enterocolitis	PO	NA	9 days	Seizures, visual impairment, altered mental status, ataxia, dysarthria	splenium of the corpus callosum, cerebral white matter	NA	Near complete resolution	Resolution
2020, Italy (10)	0.4/M	Postoperative prophylaxis after intestinal surgery	PO, IV	NA	NA	Seizures	Dentate nucleus, splenium of the corpus callosum, cerebellar white matte, cerebral white matter	40 days	Improvement	Resolution
2020, Iran (12)	11/M	Febrile bloody diarrhea	PO, IV	12	NA	Hearing loss, altered mental status, extrapyramidal symptoms, seizures, dysarthria, dysphagia	Dentate nucleus, splenium of the corpus callosum, basal ganglia, midbrain, cerebral white matter	7 months	Residual signal intensities with cystic change in basal ganglia and mild atrophic changes	residual extrapyramidal symptoms
2020, USA (11)	8/M	Postoperative prophylaxis after small bowel transplantation	NA	1378.8	3 years	Ataxia	Dentate nucleus, splenium of the corpus callosum, medulla oblongata, basal ganglia	3 weeks	Resolution	Resolution
2019, USA (14)	11/M	Fusobacterium meningitis	PO	NA	3 months	Vertigo, ataxia, peripheral neuropathy	Dentate nucleus, pons, medulla oblongata	6 weeks	Resolution	Resolution
2018, USA (13)	17/M	Postoperative prophylaxis after bowel resection transplantation	NA	NA	NA	Ataxia	Dentate nucleus, pons, medulla oblongata	4 days	Improvement	Resolution
2016, India (15)	14/M	Acute abdominal pain	NA	NA	3 days	Altered mental status, dysarthria, seizures, peripheral neuropathy	Dentate nucleus, midbrain, medulla oblongata, cervical spinal cord, cerebellar peduncle, optic tract	NA	NA	Resolution
2015, USA (16)	36 days/M	Utero exposure	NA	NA	NA	Intermittent hypothermia, bradycardia, growth retardation, reduced activity, decreased muscle strength and tone	Dentate nucleus	NA	NA	Resolution
2013, Turkey (17)	3/M	Amoebiasis diarrhea	NA	NA	14 days	Visual impairment, dizziness, ataxia	Normal	NA	NA	Resolution
2010, USA (18)	15/F	Crohn disease	NA	NA	7 days	Ataxia	Dentate nucleus	NA	Resolution	Resolution
2007, USA (19)	Teenager/M	Appendicitis	IV	NA	22 h	Altered mental status	Cerebral white matter	NA	NA	Resolution
2002, USA (20)	17/M	Crohn's disease	NA	NA	NA	Visual impairment, ataxia, peripheral neuropathy	Midbrain, basal ganglia, splenium of the corpus callosum, thalamus	3 months	Near complete resolution	Resolution
1983, USA (21)	12/M	Appendicitis	IV	4	4 days	Seizures, altered mental status	NA	NA	NA	Resolution

F, female; M, male; NA, not available; PO, per oral; IV, intravenous; PR, per rectum.

## 4 Discussion

Metronidazole is a nitroimidazole antibiotic widely used for treating anaerobic bacterial and protozoal infections. Although its overall safety profile is favorable, it is associated with rare but serious peripheral and central nervous system complications (22). The mechanisms underlying metronidazole neurotoxicity remain incompletely elucidated, with several hypotheses proposed. Some theories suggest that it functions as a thiamine analog, potentially interfering with cellular energy metabolism through inhibition of thiamine pyrophosphorylation (23). Alternatively, it might bind to neuronal RNA, thereby inhibiting protein synthesis and inducing cellular dysfunction. Furthermore, its reported ability to oxidize neurotransmitters generates neurotoxic semiquinone radicals which cause oxidative damage (24). MIE is a central nervous system toxicity of metronidazole, classically presenting with cerebellar dysfunction, altered mental status, and seizures. MIE typically exhibits a reversible course, with most patients achieving full symptom resolution following timely drug discontinuation (1, 2). However, delayed diagnosis may lead to permanent sequelae and an increased risk of mortality.

A systematic review of MIE indicates common clinical indications for metronidazole include treatment and prophylaxis of gastrointestinal infections, hepatic abscesses, and central nervous system infections. Reported treatment durations preceding MIE onset range from 2 days to 8 years (median 28 days). The cumulative dosage ranges from 5 to 2,000 g (median 65.4 g) (1). These data indicate substantial variability in both treatment duration and cumulative metronidazole exposure. MIE can occur early in the course of treatment as well as at low cumulative doses. Currently, there is no evidence that the metronidazole treatment duration or cumulative dose could predict the risk of MIE. In our case, the patient received intravenous metronidazole for CDAD. The first treatment course lasted 39 days (cumulative dose 31.2 g), with no neurological complications observed. Following a 44-day discontinuation period, metronidazole was re-administered for 47 days (cumulative dose 37.6 g), when neurological symptoms developed. Metronidazole is primarily metabolized by the liver, and its metabolites are excreted renally. This patient had abnormal liver function, which likely contributed to reduced metronidazole metabolism and subsequent drug accumulation. Thus, it may serve as a key risk factor for MIE development in this patient. This further underscores the critical importance of enhanced monitoring for neurotoxic effects and ensuring optimal metabolic clearance when using metronidazole in patients with hepatic impairment.

The clinical manifestations of MIE are diverse. Typical symptoms include cerebellar dysfunction, such as dysarthria, gait instability, ataxia, altered mental status, and seizures. Additionally, it may present as dizziness/vertigo, dystonia, hearing loss, visual impairment, and even complicated by peripheral neuropathy (6). Our patient presented with severe manifestations. Initial symptoms included irritability, dysarthria, and involuntary movements such as tongue protrusion, blinking, and grimacing. Subsequently, she developed lethargy, aphasia, dysphagia, dystonia, urinary retention, external ophthalmoplegia, and chemosis. Neurological examination showed muscle weakness and absent tendon reflexes. In summary, this patient had extensive nervous system involvement, including cortical involvement presented as encephalopathy, extrapyramidal symptoms of dystonia and involuntary movements, brainstem involvement presented as external ophthalmoplegia and dysphagia, autonomic nerve involvement presented as urinary retention, and peripheral nerve involvement manifested as muscle weakness and absent tendon reflexes.

Neuroimaging plays a pivotal role in diagnosing MIE. Characteristic brain MRI lesions include symmetrical T2W/FLAIR hyperintensities. These lesions mainly involve the dentate nuclei of the cerebellum, midbrain, and splenium of the corpus callosum, and also involve the dorsal pons, medulla oblongata, basal ganglia, thalamus, cerebellar white matter, and cerebral white matter (25). Restricted diffusion is frequently present within these lesions, but the ADC values are variable, exhibiting low, normal, or high signals. Typically, ADC signals in lesions of the corpus callosum and cerebral white matter are low while those are uncertain in dentate nuclei and other regions. This difference in ADC signal suggests that metronidazole neurotoxicity may involve both cytotoxic and vasogenic edema mechanisms. Our patient had the characteristic MRI changes, and there was significant radiological improvement after metronidazole withdrawal (Figure 2).

MIE requires differentiation from Wernicke's encephalopathy, hepatic encephalopathy, other toxic encephalopathies, and metabolic encephalopathies such as biotin-thiamine-responsive basal ganglia disease (BTBGD) and Leigh syndrome. Distinguishing MIE from Wernicke's encephalopathy is particularly crucial (24). Wernicke's encephalopathy classically presents with the triad of ophthalmoplegia, mental status changes, and ataxia. Characteristic brain MRI lesions involve the thalamus, hypothalamus, mammillary bodies, periaqueductal area, tectal plate of the fourth ventricle, and superior cerebellar vermis (26). A detailed medical history, medication history and brain MRI lesions are essential for differential diagnosis. Given metronidazole's potential role as a thiamine antagonist, its clinical manifestations and imaging changes bear a striking resemblance to those of BTBGD. However, BTBGD results from pathogenic variants in the SLC19A3 gene (7). Therefore, besides medication history, genetic mutation analysis serves as a key differentiating tool.

The treatment of MIE lies in the timely discontinuation of metronidazole. Given that metronidazole may serve as a thiamine antagonist and interfere with its metabolism, routine thiamine supplementation has been suggested as adjunctive therapy. Most cases of MIE are reversible, with clinical and imaging improvements after metronidazole withdrawal, but a few cases may leave neurological sequelae (27). In this patient, metronidazole was immediately discontinued and vitamin B1 was added after considering MIE. Her encephalopathic manifestations gradually improved 4 days after cessation. Follow-up brain MRI at 10 days after drug withdrawal demonstrated partial resolution of signal abnormalities, while extrapyramidal symptoms persisted. Due to the delayed diagnosis, the neurological involvement in the patient has become more extensive, and the extrapyramidal symptoms failed to resolve after drug withdrawal. Regrettably, approximately one month after metronidazole withdrawal, the patient succumbed to acute gastrointestinal hemorrhage secondary to GVHD.

In summary, MIE is rare in children but largely reversible. A history of metronidazole exposure and characteristic brain MRI lesions facilitate diagnosis. Prompt recognition of MIE and immediate drug discontinuation constitute the cornerstone of management. Moreover, heightened vigilance for potential neurotoxicity is imperative when administering metronidazole to pediatric patients with hepatic or renal impairment.

## Data Availability

The datasets presented in this article are not readily available because of ethical and privacy restrictions. Requests to access the datasets should be directed to the corresponding author.
